# Community’s knowledge, attitudes and practices about tuberculosis in Itang Special District, Gambella Region, South Western Ethiopia

**DOI:** 10.1186/1471-2458-13-734

**Published:** 2013-08-07

**Authors:** Jango Bati, Mengistu Legesse, Girmay Medhin

**Affiliations:** 1 Gambella Teachers Education and Health Science College, Gambella, Ethiopia; 2 Addis Ababa University, Aklilu Lemma Institute of Pathobiology, Addis Ababa, P.O. Box, 1176, Ethiopia

## Abstract

**Background:**

Tuberculosis (TB) is one of the primary public health problems in developing countries. HIV/AIDS, poverty, undernutrition, over-crowded living conditions and lack of knowledge about the disease have been known to increase the risk of spreading the bacteria and the risk of developing the disease. The objective of this study was to assess the level of TB knowledge, attitudes and practices of rural communities of Itang Special District of the Gambella Regional State of Ethiopia.

**Methods:**

Between November 2011 and January 2012, a community-based cross sectional study was carried out in a randomly selected rural kebeles (i.e. the smallest administrative units) of Itang communities. The study participants were interviewed using pre-tested questionnaire. The overall knowledge, attitudes and practices of the study participants were assessed using the mean score of each outcome as a cut-off value. Having a score above the mean on each of the three target outcomes was equated with having a good level of knowledge, or having favorable attitude and good practices towards TB.

**Results:**

Out of 422 study participants (58.5% males and 41.5% females) only 3.3% mentioned bacteria/germ as a cause of pulmonary TB (PTB) and 9.9% mentioned cough for at least two weeks as the sign of TB. Taking the mean knowledge score as the cut-off value, 57.6% (95% CI: 52.7% to 62.3%) of the study participants had good level of knowledge about TB, 40.8% (95% CI: 36.0% to 45.6%) had favorable attitude towards TB and 45.9% (95% CI: 41.1% to 50.9%) had good practices. Female participants were less likely to have good level of knowledge [adjusted odds ratio (AOR) = 0.33, 95% CI, 0.21 to 0.51, p < 0.001], less likely to have favorable attitude (AOR = 0.23, 95% CI, 0.14 to 0.37) and less likely to have good practices (AOR = 0.37, 95% CI, 0.24 to 0.57, p < 0.001) compared to male participants.

**Conclusion:**

Majority of the study participants had no correct information about the causative agent of TB and the main symptom of PTB. Moreover, low level of overall knowledge, attitudes and practices about TB was associated with female participants. Hence, TB control strategy in the present study area should include community awareness raising component.

## Background

Tuberculosis (TB) is a chronic bacterial disease mainly caused by *Mycobacterium tuberculosis (M.tb)*[1]. The disease is mainly transmitted through air droplets, cough, and sneeze, when patient expels droplets containing bacilli and inhaled by healthy individuals. Although TB primarily affects the lungs it also affects any part of the body including kidney, brain, intestine, bone and lymph nodes [2].

Each year, an individual with active Pulmonary Tuberculosis (PTB) infects an average of 10–15 people [3]. Moreover, in a community with low levels of awareness about the cause, mode of transmission and preventive methods, the spreading of TB could be high [4,5]. The estimates for the year 2010 global TB prevalence and incident cases were 12 and 8.8 million respectively [2]. The vast majority of the cases (95%) and deaths (98%) occur in resource limited countries [3]. This situation has been aggravating with growing problem of resistances to first line anti-TB drugs resulting in half million of new cases of multi drug resistances (MDR TB) [6,7]. Therefore, TB situation is most likely to continue to deteriorate due to multiple factors such as HIV/AIDS pandemic, poverty, low level of awareness and adverse effect of poor quality TB program [3].

Ethiopia is among the 22 High Burden Countries (HBCs) [8] and TB accounts for the major proportion of hospital admissions and reported as being the third leading cause of hospital deaths next to malaria and obstetric cases [9]. In the year 2010,the national estimate of the prevalence and incidence and mortality rates of TB were 294, 261 and 35 per 100,000 population respectively, whereas smear-positive PTB (SPPTB) cases were estimated to be 46,634 among adults aged above 15 years [2]. To reverse the impact of TB situation, TB control strategies including decentralizing/expansion of TB diagnosis and treatment services to peripheral units like health posts, private clinics, expanding health extension program (HEP) and engagement of communities through health extension workers (HEWs) have been the focus of Federal Ministry of Health [10]. One study in the southern part of Ethiopia indicated that engagement of the HEWs has improved TB case detection rate (CDR), treatment success rate (TSR), community’s awareness in TB suspect identification/contact tracing activities and screening of TB patients for HIV infection [11].

Gambella Regional State (GRS) is one of the high TB burden regional sates within Ethiopia [3]. In the year 2010, 953 TB cases were detected and treated in this Regional State and among these cases 621 patients were PTB cases [12]. Even though assessment of communities’ knowledge, attitude and practice (KAP) are key elements in prevention and control of TB parallel to other control strategies [3], there is no/little information on communities’ KAP towards the control of TB in GRS. The objective of this study was to assess communities’ KAP regarding TB in Itang Special District of the GRS, South Western Ethiopia.

## Methods

### Study area and population

The current study was conducted in Itang Special District of the GRS, South Western Ethiopia. Itang Special District is located at 822 km south west of Addis Ababa. The region shares its boards with Oromia Regional State in the North and East, Southern Nations, Nationalities and Peoples Region (SNNPRS) in the South and in the East, and the Republic of South Sudan in the West. The major ethnic groups in the region includes; Anywa, Nuer, Mezengir, Opwo and Komo. According to the 2007 housing and population census of Ethiopia [13], the region has a total population of 306,916, consisting of 159,679 males and 147,237 females. Among the residents of the region the proportion of urban inhabitants is 25%.

Recession river side agriculture is common in the region. In particular, maize and sorghum are widely practiced by Anywa and Opwo, whereas livestock constitutes the primary sources of income for Nuer community. The region has one hospital which gives routine service for urban population and provided referral service for rural people. In addition to this hospital, there are 22 health centers, 34 clinics, 108 health posts, and 16 drug venders in the region [12-14].

Itang Special District was purposely selected for the current study from the 14 districts of the region because most of its residents are indigenous and live in the remotest area of the region compared to other districts. The district has a total of 21 kebeles (small administrative units). Based on the 2007 Ethiopian National population and housing census, it has a total population of 41, 463, while rural population comprises 82%.

The main indigenous ethnic groups of the District are Nuer (61%), Anywa (30%) and Opwo (8.5%), and they live in stratified manner of settlements based on the ethnic group. Health service coverage is estimated to be 60.3% with 3 health centers, among which only one health center has been providing TB services for the district. In addition, there are 8 health posts in the rural areas among which only 5 have been providing health services. Malaria and TB are among the top diseases in the District [12,14].

### Study design, sampling and data collection

Between November 2011 and January 2012, a community-based cross-sectional study was conducted in a randomly selected eight rural kebeles. Prior to data collection, the study kebeles were stratified in to 3 strata based on how the areas are populated by different indigenous ethnic groups (i.e. Nuer, Anywa, and Opwo). Anywa site contains 11 kebeles with a total households of 2497, Nuer site contains 8 kebeles with a total households of 5537 and Opwo site contains only one kebele with a total households of 275. Based on the number of kebeles in each strata, 4 kebeles from Anywa site (Adeng, Awanyi, Elia and Itangkir), and 3 kebeles from Nuer site (Achuwa, Mokod and Watgach) were randomly selected and the only kebele in the Opwo site (i. e. Puldeng) was included in the study. The number of households to be included in the study was determined using the formula appropriate for estimation of single proportion. As an input we assumed that, the overall level of good knowledge of the community about TB is 50% with 95% confidence in the estimate, precision of 5% and 10% of none response rate. Hence, the minimum required sample size was 422 respondents. The list of the total households of each study kebele was obtained from respective health extension workers (HEWs). Pre-determined sample size of 422 was proportionally allocated to each kebele based on the number of households within each kebele. Respondents from the target kebeles were selected using systematic random sampling taking the list of households as the sampling frame.

Data were collected using pre-tested structured questionnaires adopted from previous studies [15,16]. The questionnaires were cheeked for clarity, consistence and cultural acceptability in adjacent kebeles where the actual study didn’t take place. The questionnaires were prepared in English and asked directly by translating into the local languages orally (Nuer, Anywa, and Opwo). A total of three trained diploma level nurses who are fluent in speaking and writing of indigenous language were participated in the data collection.

### Ethical considerations

The study protocol was approved by the Institutional Review Board (IRB) of the Aklilu Lemma Institute of Pathobiology (ALIPB), Addis Ababa University and permission was obtained from Gambella Regional Health Bureau. The objectives of the study were explained to the study participants and verbal consent was obtained before interviewing each participant.

### Data analysis

The collected data was computerized using Epi Data software Version 3.1 and exported into STATA version 11. Pearson chi-square test, univariable logistic regression analysis and multivariable logistic regression analysis were performed to explore the association between outcomes and predictor variables. Overall knowledge of the study participants about TB was assessed using six major questions and 35 sub-questions, such as source of information about TB, able to mention cause of TB, sign and symptoms of TB, mode of transmission of TB, risk factors for TB, and identifying individuals at high risk for TB. For each question, a score of one was given to correct response and score of zero was given to the “do not know” response and incorrect answers. The overall knowledge score was obtained by summing these responses which is expected to range between 0 and 35. The composite score was dichotomized using mean obtained from the data (i.e. mean = 15.2). Individuals who have scored above and equal to the mean were categorized as having good level of knowledge and those who have scored below the mean knowledge score were classified as having poor level of knowledge. Similar procedures were used in defining attitude and practices as outcome variables by asking 11 and 13 questions, respectively. The questions were mainly on stigmatizing patients, wrong perceptions and what communities do before and after suffering from TB. Then, mean value of 3.2 for attitude and the mean value of 3.4 for practice were used to categorize respondents into groups having favorable versus non-favorable attitude and good practices versus poor practices, respectively.

## Results

### Socio-demographic characteristics of the study participants

A total of 422 study participants (58.5% males and 41.5% females; age ranged from 19-81 years) were interviewed and the response rate was 100%. The mean age of the study participants was 37 years (SD =11.6). About half (50.7%) were illiterates and 70.9% were pastoralists (Table 1). With regard to ethnic composition, 127 (30.1%) were Anywa, 281 (66.6%) were Nuer, and 14 (3.3%) were Opwo. Majority of the study participants (78.4%) were followers of Protestant religion, 11.4% were Seventh day followers, 56.8% had at least 5 children and 83.7% of the respondents were married.

**Table 1:** Socio-demographic characteristics of the study participants and communities’ overall knowledge of TB, Itang Special District, South Western Ethiopia

**Characteristics**	**Total number (%) interviewed**	**Level of knowledge score (max 35) Good (Score ≥15.2) poor score**		**COR (CI)**	**AOR (CI)**
Sex					
Males	247 (58.5)	172 (69.6%)	75 (30.4%)	1	1
Females	175 (41.5)	71 (40.6%)	104 (59.4%)	0.29 (0.19, 0.44)	0.33 (0.21, 0.51)
**Marital status**					
Married	353 (83.7)	197 (55.8%)	156 (44.2%)	1	1
Others	69 (16.3)	46 (66.7%)	23 (33,3%)	1.58 (0.92, 2.73)	2.08 (1.02, 4.27)
**Age categories**					
19–29	122 (28.9)	66 (54.0%)	56 (46.0%)	1	1
30–44	198 (46.9)	131 (66.2%)	67 (33.8%)	1.65 (1.04, 2.63)	3.13 (1.75, 5.61)
45–59	77 (18.3)	39 (50.6%)	38 (49.4%)	0.87 (0.49,1.54)	1.93 (0.91, 4.09)
≥60	25 (5.9)	7 (28.0%)	18 (72.0%)	0.32 (0.12, 0.84)	0.78 (0.26, 2.32)
**Sites**					
Anywa	127 (30.1)	106 (83.5%)	21 (16.5%)	1	1
Nuer	281 (66.6)	125 (44.5%)	156 (55.5%)	0.15 (0.09, 0.26)	0.205 (0.11, 0.36)
OPwo	14 (3.3)	12 (85.7%)	2 (14.3%)	1.18 (0.24, 5.70)	1.63 (0.32, 8.18)
**Education**					
Illiterate	214 (50.7)	116 (54.2%)	98 (45.8%)	1	1
Read & write	59 (14.0)	33 (55.9%)	26 (44.1%)	1.07 (0.60, 1.91)	0.85 (0.43, 1.67)
Primary school	96 (22.8)	52 (54.2%)	44 (45.8%)	0.99 (0.61, 1.61)	0.854 (0.43, 1.67)
Secondary school and above	53 (12.6)	42 (79.3%)	11 (20.8%)	3.22 (1.57, 6.60)	3.076 (1.08, 8.68)
**Occupation**					
Pastoralist	199 (70.9)	160 (53.2%)	139 (46.5%)	1	1
Merchant	13 (3.1)	7 (53.5%)	6 (46.6%)	1.01 (0.33, 3.08)	0.65 (0.19, 2.13)
Students	39 (9.2)	29 (74.4%)	10 (25.6%)	2.519 (1.18, 5.35)	1.41 (0.57, 3.49)
Employees	71 (16.8)	47 (66.2%)	24 (33.8%)	1.70 (0.98, 2.92)	1.38 (0.61, 3.08)

*COR* crude odd ratio, *AOR* adjusted odd ratio.

### Knowledge about the cause and signs/symptoms of TB

Information on the knowledge of respondents about cause and symptoms of TB is summarized in Table 2. Most of the respondents (94.3%) have heard about TB. The sources of information for the respondents were HEWs (41.9%), TB patients (37.7%), friends/relatives (34.4%), school (15.6%), public radio (0.95%) and television (1.6%). Alcohol consumption (42.7%), exposure to cold air (16.6%), germ/bacteria (3.3%) and smoking (13.9%) were regarded as primary causes of PTB by the respondents. The primary cause of tuberculosis lymphadenitis (TBL) was mentioned as exposure to cold air (26.1%), smoking (17.5%) and drinking cows’ raw milk (16.6%). Hemoptsis (60.2%), chest pain (30.8%), weight loss (14.7%) and cough for two weeks and above (9.9%) were mentioned as a major signs and symptoms of PTB.

**Table 2:** Communities’ knowledge about the cause, sign and symptoms of TB, Itang Special District, GRS, South Western Ethiopia

**Variable**	**Response categories**	**Anywa site**	**Nuer site**	**Opwo site**	**Total %**
**Cause of PTB**		Number (%)	Number (%)	Number (%)	Number (%)
	Bacteria	8 (6.3)	6 (2.1)	0 (0.0)	14 (3.3)
	Cold air	8 (6.3)	51 (18.2)	11 (78.6)	70 (16.6)
	Hot climate	1 (0.8)	5 (1.8)	0 (0.0)	6 (1.4)
	Shortage of food	17 (13.4)	4 (1.4)	1 (7.1)	22 (5.2)
	Alcohol	81 (63.8)	86 (30.6)	13 (92.8)	180 (42.7)
	Smoking	14 (11.1)	40 (14.2)	5 (35.7)	59 (13.9)
	Don’t know	4 (3.2)	143 (50.8)	4 (3.2)	151 (33.6)
**Cause of (TBL)**	Bacteria	4 (3.2)	5 (1.8)	0 (0.0)	9 (2.1)
	Cold air	45 (35.4)	58 (20.6)	7 (50.0)	110 (26.1)
	Smoking	42 (33.1)	30 (10.7)	2 (14.3)	74 (17.5)
	Drinking cow raw milk	40 (31.5)	27 (9.6)	3 (21.4)	70 (16.6)
**Signs and symptoms of PTB**	Cough for 2 weeks & above	29 (22.8)	10 (3.6)	3 (21.4)	42 (9.9)
	Weight loss	35 (27.6)	20 (7.1)	7 (50.0)	62 (14.7)
	Hemoptsis	81 (63.8)	176 (59.4)	6 (42.8)	254 (60.2)
	Chest pain	60 (47.2)	69 (24.5)	1 (7.1)	130 (30.8)
	Don’t know	3 (2.4)	88 (31.3)	0 (0.0)	91 (19.2)

### Knowledge about the mode of transmission and preventive methods of TB

Respondents’ responses regarding mode of transmission and preventive methods of TB are summarized in Table 3. About half (51.1%) of the study participants said that PTB is transmit through cough and 31.3% said that both PTB and TBL can be transmitted through sharing of drinking materials. About 15% of the participants mentioned that they vaccinate their children with BCG to protect PTB and TBL. This practice was more frequent among Nuer ethnic group compared to Anywa ethnic group (22.8% versus 0.8%, p < 0.001). Hygiene was mentioned as a means of preventing both PTB and TBL by 29.2% of the study participants. Forty one percent of the study participants mentioned “avoiding of smoking” as the most important method of preventing and control of PTB. About 90% of the study participants said that PTB is treatable with modern drugs, whereas 37.4% said that they treat TBL by burning the swelling sites with heated metal materials culturally.

**Table 3:** Communities' knowledge about mode of transmission and preventive methods of TB, Itang Special District, GRS, South Western Ethiopia

**Variables**	**Anywa site**	**Nuer site**	**Opwo site**	**Total**
Is PTB transmittable?	Number (%)	Number (%)	Number (%)	Number (%)
Yes	114 (89.8)	219 (77.9)	13 (92.8)	346 (81.9)
No	13 (10.2)	62 (22.1)	1 (7.1)	76 (16.2)
**Mode of TB transmission**				
Transmits through cough (PTB)	65 (51.1)	142 (50.5)	10 (71.4)	272 (51.4)
Through sharing drinking (TBL and PTB)	46 (36.2)	76 (27.1)	10 (71.4)	132 (31.3)
Through sharing food (TBL and PTB)	18 (14.2)	69 (24.5)	10 (71.4)	97 (22.9)
**Prevention and control of TB**				
BCG vaccination (PTB and TBL)	1 (0.8)	64 (22.8)	0 (0.0)	65 (15.4)
Hygiene (PTB and TBL)	28 (22.1)	90 (32.1)	5 (35.7)	123 (29.2)
Avoid smoking (PTB)	87 (68.5)	76 (27.1)	10 (71.4)	173 (41.0)
Isolate patient with PTB	16 (12.5)	8 (2.9)	5 (35.7)	29 (6.8)
Cover mouth when cough for PTB	21 (16.5)	2 (0.7)	7 (50.0)	30 (7.1)
Avoid drinking raw milk for TBL	51 (40.2)	21 (7.5)	8 (57.1)	80 (18.9)
Consult health extension workers incase sick from PTB	95 (74.8)	115 (40.9)	11 (78.6)	221 (52.4)
Prefer traditional healers for treatment (TBL)	33 (25.9)	157 (55.8)	1 (7.2)	191 (45.3)
Prefer health post for treatment (PTB)	33 (25.9)	143 (50.9)	4 (28.5)	180 (42.7)

Twenty two percent of the study participants reported that at least one of their families’ members had previous history of PTB and treated with modern drugs at health centers. About half of the study participants prefer to consult CHEWs in case they and their families sick from PTB. However, participants from Nuer site were less likely to consult CHEWs compared to participants from Opwo site (40.9% versus 78.6%, P < 0.001). Forty five percent of the participants preferred health center for the treatment of major sign/symptoms of PTB, whereas 42.7% of the participants preferred to be treated for major sign/symptoms of PTB in health posts.

### Communities’ overall knowledge about TB

The level of overall knowledge generated using the composite knowledge score is summarized in Table 1. Of the total study participants 243 (57.6% 95% CI, 52.7% to 62.3%) had good level of knowledge about TB. Female participants (adjusted odd ratio, AOR = 0.33, 95% CI, 0.21 to 0.51, p < 0.002) and participants from Nuer site (AOR = 0.21; 95% CI: 0.11 to 0.36) had low level of overall knowledge about TB. Being in the age categories of 30–44 years compared to 19–29 years of age group (AOR = 3.14, 95% CI, 1.76 to 5.62, p < 0.001) and being relatively well educated than being illiterate (AOR = 3.08, 95% CI, 1.09 to 8.68, p = 0.001) had good level of overall knowledge about TB.

### Communities’ attitude/perception towards TB

Summary information about the attitude of study participants about TB is summarized in Table 4. From the total of 422 study participants, 263 (58%) mentioned PTB as killer disease even after treatment, whereas 138 (34.1%) said that it affects only poor people. Thirty (7.1%) respondents reported that they don’t share food and drink with TB patient, while 257 (59%) said that TB patients should feel shame which was highly reflected among participants from Nuer site (85.8%). About 40.8% (95%CI, 36.0% to 45.6%) of the study participants had favorable attitude towards TB. Low favorable attitude was significantly associated with female gender (AOR = 0.23, 95% CI, 0.14 to 0.37). Favorable attitude towards TB patients was significantly associated with high educational level of the study participants (AOR = 4.78, 95% CI, 1.78 to 12.83). Occupation of the participants other than pastoralist were also associated with favorable attitude towards TB (AOR = 5.92, 95% CI, 3.53 to 9.92).

**Table 4:** Communities’ attitude towards TB, rural of Itang Special District, GRS, South Western Ethiopia, 2012

**Characteristics**	**Level of attitude**		**Crude OR (CI)**	**AOR (CI)**
**Sex**	Favorable ≥3.2	Unfavorable <3.2		
Males	128 (51.8%)	119 (48.2%)	1	1
Females	44 (25.1%)	131 (74.9%)	0.31 (0.20, 0.48)	0.22 (0.13, 0.37)
**Age categories**				
(19–29)	63 (51.6%)	59 (48.4%)	1	1
(30–44)	74 (37.4%)	124 (62.4%)	0.55 (0.35, 0.88)	1.35 (0.77, 2.37)
(45–59)	29 (37.6%)	48 (62.4%)	0.87 (0.49, 1.54)	1.93 (0.91, 4.09)
≥60	6 (24.0%)	19 (76.0%)	0.32 (0.12, 0 .87)	0.78 (0.26, 2.32)
**Sites**				
Anywa	45 (35.4%)	82 (64.5%)	1	1
Nuer	115 (40.9%)	166 (59.1%)	1.26 (0.82, 1.95)	1.49 (0.82, 2.23)
OPwo	12 (85.7%)	2 (14.3%)	10.93 (2.34, 51.02)	13.30 (2.77, 63.96)
**Educational status**				
Illiterate	62 (28.9%)	152 (71%)	1	1
Read & write	24 (40.7%)	35 (59.3%)	1.68 (0.92, 3.05)	1.26 (0.64, 2.49)
Primary school	49 (51%)	47 (48.9%)	2.55 (1.55, 4.20)	2.26 (1.15, 4.45)
Secondary school & above	37 (69.8%)	16 (30.2%)	5.66 (2.94, 10.93)	4.77 (1.77, 12.83)
**Occupational status**				
Pastoralist	96 (32.1%)	203 (67.9%)	1	1
Merchant	8 (61.5%)	5 (38.5%)	3.38 (1.07, 10.61)	3.88 (1.00, 15.02)
Students	25 (64%)	14 (35.9%)	3.77 (1.87, 7.58)	3.522 (1.51, 8.17)
Employees	43 (60.6%)	28 (39.4%)	3.24 (1.90, 5.54)	3.554 (1.94, 6.49)
**Knowledge**				
Poor	179 (56.6%)	141 (43.4%)	1	1
Good	243 (57.6%)	109 (42.4%)	4.78 (2.81, 8.15)	5.92 (3.53, 9.92)

### Communities’ overall practices regarding prevention and control of TB

Information about the overall practice of the communities is summarized in Table 5. Less than half of the participants (45.9% 95% CI, 41.1% to 50.9%) had good practices. Poor practices of prevention and control of TB was significantly associated with female participants (AOR = 0.37, 95% CI, 0.24 to 0.57, p < 0.001).

**Table 5:** Communities’ practices towards TB prevention and control, Itang Special District, GRS

**Characteristics**	**Level of practices**		**Crude OR (CI)**	**AOR (CI)**
	Good ≥3.4	Poor <3.4		
**Sex**				
Males	138 (55.8%)	109 (44.2%)	1	1
Females	56 (32%)	119 (68%)	0.37 (0.24, 0.55)	0.36 (.24, 0.56)
**Marital status**				
Married	154 (43.6%)	199 (56.4%)	1	1
Others	40 (57.9%)	29 (42.0%)	1.78 (1.05, 3.00)	1.61 (0.93, 2.80)
**Age categories**				
(19–29)	60 (49. 2%)	62 (50.2%)	1	1
(30–44)	92 (46.5%)	106 (53.2%)	0.89 (0.57, 1.40)	1.33 (0.78, 2.25)
(45–59)	34 (44.5%)	43 (55.8%)	0.81 (0.46, 1.44)	1.54 (0.77, 3.11)
≥60	8 (32.0%)	17 (68.0%)	0.48 (0.19, 1.21)	1.04 (0.37, 2.88)
**Sites**				
Anywa	74 (58.3%)	53 (41.7%)	1	1
Nuer	107 (38.1%)	174 (61.9%)	0.44 (0.28, 0.67)	0.58 (0.35, 0.96)
OPwo	13 (92.9%)	1 (7.1%)	9.31 (1.18, 73.36)	14.5 (1.78, 11.85)
**Educational status**				
Illiterate	89 (41.5%)	125 (58.4%)	1	1
Read & write	29 (49.2%)	30 (50.5%)	1.35 (0.76, 2.42)	1.17 (0.62, 2.23)
Primary school	45 (46.9%)	51 (53.1%)	1.23 (0.76, 2.01)	0.93 (0.48, 1.76)
Secondary school & above	31 (58.9%)	22 (41,5%)	1.97 (1.07, 3.64)	1.15 (0.46, 2.82)
**Occupational status**				
Pastoralist	122 (40.8%)	177 (59.2%)	1	1
Merchant	7 (53.9%)	6 (46.2%)	1.69 (0.55, 5.15)	1.58 (0.47, 5.26)
Students	24 (61.5%)	15 (38.5%)	2.32 (1.17, 4.61)	2.31 (1.01, 5.30)
Employees	41 (57.8%)	30 (42.3%)	1.98 (1.17, 3.34)	2.25 (1.05, 4.82)
**Knowledge**				
Poor	179 ((59.6%)	147 (40.4%)	1	1
Good	243 (64.6%)	81 (35.4%)	8.52 (5.07, 14.32)	9.64 (5.76-16.01)

## Discussion

The results of this community-based cross-sectional study showed that most of the community’s members of Itang Special District have information about TB. The finding is similar to the results of studies from Afar Region [16], South West Ethiopia [17] and North Ethiopia [15,18], Malaysia [19] and rural China [20]. The main sources of information mentioned by the study participants are also similar to the findings of study conducted in other part of Ethiopia [15]. In the current study, very few participants mentioned mass media as a source of information about TB which is inconsistent with studies done in Lahore [21], Iraq [22] and Pakistan [23] in which most of the study participants’ mentioned mass media as a major source of information about TB.

Despite a higher proportion of the study participants have heard about TB, the majority had no/little knowledge about the cause of the disease. Only 3.3% of the participants correctly answered the cause of TB as being germ/bacteria which is in agreement with the findings of study from other part of Ethiopia [16]. Cold air, alcohol consumption, smoking and shortage of food were frequently mentioned as the cause of PTB by the present study participants which is similar to the findings of studies in Tigray, Afar and North Ethiopia [15,16,18], but differ from the findings of previous study on TB suspects in a rural community of southwest Ethiopia, in which sizeable number of the study participants mentioned bacteria as the cause of TB [17]. The difference might be due to early deployment of HEWs in the previous study area as compared to the present study area [9]. In addition to this, a rural community of South West Ethiopia has been exposed to students’ practicum program from Medical school of the Jimma University. Hence, relatively information on TB might be easily disseminated among that community as compared to the present studied community [24].

In previous studies conducted in varies parts of Ethiopia [15-18], the majority of the study participants identified persistent productive cough as the major signs and symptoms of PTB. In the present study, persistent productive cough was mentioned as signs and symptoms of PTB by very few study participants. The discrepancies might be due to socio-cultural factors such as smoking of Gaya by most of the indigenous in the present study area [12].

Gaya is a local name given to instrument used to prepare and suck after tobacco, few drops of water is added and roasted with fire gently until evaporation starts to make it comfortable for sucking. It causes addiction like any other addiction causative substances such as cigarette. It causes productive cough among smokers. This situation misleads perception of the communities in that, productive cough is caused by smoking of Gaya rather than due to PTB and delay them from early diagnosis and treatment of PTB. Results of the present study regarding the signs and symptoms of PTB are also inconsistent with the findings of other studies conducted elsewhere [20,21] in which majority of the participants mentioned persistent productive cough as major signs and symptoms of PTB.

In this study, the study participants reported that PTB is treatable and curable with modern drug which is consistence with the findings of previous studies in other parts of Ethiopia [15,16], in Iraq [22], in South Africa [25] and in Tajikistan [26]. However, the finding of the current study is different from the results of previous studies conducted in south part of Ethiopia [27] and in Tanzania in which greater than half of the study participants reported roots bark from trees, oil from meat, blood, and fat as the effective treatment for TB [28]. High level of awareness of the communities about the appropriate treatment of the disease could have significant implications in reducing diagnosis and treatment delay, as well as the spread of the disease.

The present study showed that a considerable proportion of the study participants had unfavorable attitude towards TB patients which could potentially create ground for stigmatization of TB patients in the communities [18,25,29]. On the other hand, the participants recognized that specific groups of population/individuals such as poor, people living with HIV/AIDS, alcohol consumption and smokers are at high risk of getting TB which corroborates the findings of previous studies conducted in Ethiopia [15-17], in India [29], Vietnam [30] and Tajikistan [26]. More than half of the study participants thought that TB is killer even after treatment in patients who have low socio economic status, which is similar to the finding of study conducted in West Bengal in which the majority of the study participants including community leaders felt that poor people cannot be cured completely from TB by treatment [31].

The study participants indicated that the communities in the present study area are engaged in different practices such as cultural and modern facility based mixed approaches of treatments for TBL. A considerable proportion (45%) of the study participants said that they consult traditional healers for the treatment of TBL, and the traditional healers treat the site of swelling by burning with metal materials. Study in other part of Ethiopia indicated that patients with TBL are treated with herbal medicine by traditional healers [16]. The discrepancies in traditional ways of treatment of TBL might be aroused from several factors like cultural and environmental differences of the studied communities.

About half of the study participants’ consult HEWs for the treatment of PTB at health post levels which disagrees with findings of previous study in North part of Ethiopia [18] in which majority of the PTB suspects visited medical health providers at health centers and hospitals levels, where there are trained man power, diagnostic facilities and anti TB regimens relatively available. This difference might be due to early deployment of community’s HEWs in the previous study area [9]. In addition to this, majority of the present study communities are highly mobile as compared to the previous study which make difficult to provide health education on the medical importance of TB [14].

In this study, it was noted that, majority of the participants from the Nuer site had low level of overall knowledge; attitude and practices of TB compared to participants from Opwo and Anywa sites. This difference might be linked to mobility of the community as most of the inhabitants of Nuer are pastoralists which make it difficult to provide health education on the importance of medical care for TB by HEWs. The results of the study conducted in Malaysia showed absence of significant association between score on general knowledge and attitude towards TB with age, gender and educational levels of the study participants [19]. In the present study, it was found that female participants had low level of favorable attitude and poor practices towards the prevention of TB as compared to male participants. The finding is in agreement with previous study done in Ethiopia [15] and studies done elsewhere [29,32-34]. Hence, community based health education which focuses on pastoral community and females’ inhabitants could be important to improve their knowledge, attitude and practices of TB in the present study area.

## Conclusion

The results of this study revealed a low level of knowledge about the causative agent as well as about the main symptom of TB in the present study communities. In addition, the study showed low level of overall knowledge, unfavorable attitude and poor practices towards TB especially in participants from Nuer site and females. Hence, public health education on the cause, symptoms and mode of transmission of TB would be important towards the prevention and control of TB in the present study area.

## Competing interests

The authors have no competing interests.

## Authors’ contributions

JB designed the study, collected data, analysis and drafted the manuscript. ML participated in study design and write up. GM participated in study design, data analysis and write-up. All authors read, critically revised and approved the final manuscript. ML is the guarantor of the paper.

## Pre-publication history

The pre-publication history for this paper can be accessed here:

http://www.biomedcentral.com/1471-2458/13/734/prepub
